# A Novel Drug Design Strategy: An Inspiration from Encaging Tumor by Metallofullerenol Gd@C_82_(OH)_22_

**DOI:** 10.3390/molecules24132387

**Published:** 2019-06-27

**Authors:** Jinxia Li, Linlin Chen, Liang Yan, Zhanjun Gu, Zhaofang Chen, Aiping Zhang, Feng Zhao

**Affiliations:** 1 CAS Key Laboratory for Biomedical Effects of Nanomaterials and Nanosafety, Institute of High Energy Physics, Chinese Academy of Sciences (CAS), Beijing 100049, China; 2 College of Pharmacy, Shanxi Medical University, Taiyuan 030001, China; 3 Jiangsu Key Laboratory of Hazardous Chemicals Safety and Control, College of Safety Science and Engineering, Nanjing Tech University, Nanjing 210009, China

**Keywords:** metallofullerenol, Gd@C_82_(OH)_22_, encaging tumor, drug design, key and lock principle

## Abstract

Cancer remains a major threat to human health worldwide. Cytotoxicity has imposed restrictions on the conventional cytotoxic drug-based chemotherapy. The rapidly-developing nanomedicine has shown great promise in revolutionizing chemotherapy with improved efficiency and reduced toxicity. Gd@C_82_(OH)_22_, a novel endohedral metallofullerenol, was first reported by our research group to suppress tumor growth and metastasis efficiently without obvious toxicity. Gd@C_82_(OH)_22_ imprisons tumors by facilitating the formation of surrounding fibrous layers which is different from chemotherapeutics that poison tumor cells. In this review, the authors first reported the antineoplastic activity of metallofullerenol Gd@C_82_(OH)_22_ followed by further discussions on its new anti-cancer molecular mechanism—tumor encaging. On this basis, the unparalleled advantages of nanomedicine in the future drug design are discussed. The unique interaction modes of Gd@C_82_(OH)_22_ with specific targeted biomolecules may shed light on a new avenue for drug design. Depending on the surface characteristics of target biomolecules, nanomedicine, just like a transformable and dynamic key, can self-assemble into suitable shapes to match several locks for the thermodynamic stability, suggesting the target-tailoring ability of nanomedicine.

## 1. Introduction

According to the latest cancer statistics issued by the American Cancer Society in 2019, the death rate from all cancers combined has declined by 27% since 1991 owing to increased awareness, decreased smoking and progression made in early detection and treatment [1]. However, cancer, as a leading cause of morbidity and mortality, still remains a major threat to human health worldwide [2]. In the USA, 1,762,450 new cancer cases have been reported in 2019 and 606,880 deaths are projected to occur in 2019 [1]. Globally, an alarming increase in the incidence of all-cancer cases has been estimated from 12.7 million new cases in 2008 to 22.2 million by 2030 [3]. The large threat of cancer to human health has posed a great challenge for medical practice in cancer diagnosis and treatment.

Currently, three principle strategies available to treat cancers are still confined to surgery, chemotherapy and radiotherapy [4]. Either mechanical removal of tumors by surgery or direct destruction of malignant cells by poisoning or irradiating inevitably induces unsatisfactory outcomes. Chemotherapy, dependent on the cytotoxic effects of chemotherapeutics, has witnessed high occurrences of severe side effects due to its unselective damage both to tumor and normal tissues [5]. In some cases, patients suffer so much that chemotherapy is forced to be terminated. More importantly, multidrug resistance is another challenge confronted by chemotherapy, which significantly limits the effectiveness of chemotherapy and impedes the progress of patient prognosis [6,7]. Approximately, 90% of deaths from ovarian cancer could be attributed to multidrug resistance [8]. Despite the emergence of molecular-targeted chemotherapy with enhanced selectivity in late decades, adverse reactions are still unavoidable which, at least partially, is attributed to the diversity and complexity of target biomolecules [9,10]. Therefore, a new anti-cancer strategy is in great need to reduce or even avoid the risks of severe toxicity resulting from the traditional chemotherapy.

Encouragingly, the recent rapidly-developing nanotechnology offers great opportunities to improve the antineoplastic efficiency and simultaneously smooth away the drawbacks of traditional chemotherapy [11]. Globally, intensive research on the development of clinic-aimed nanotechnology has attracted increasing attention as an emerging hotspot of the nanoscience field. Nationally, facing the global competitions, our government has urgently encouraged the acceleration of the development of nanotechnology with substantial support and investment for the transition of the laboratory innovation to the practice, especially those promising breakthroughs in clinical applications [12].

The unique physicochemical properties of nanomaterials attractive for clinical applications, have prompted the development of nanomedicine and these properties also influence the behaviors of nanomedicine in the body [13,14]. The authors previously discussed the impacts of nanomaterials properties (size, shape, surface, et al.) on the metabolism of nanomaterials including circulation, organ-specific extravasation and clearance in vivo, which highlighted the flexible and controllable manipulation of the nanomaterials’ behaviors by modulating their physicochemical properties [15]. Moreover, nano-scaled size endows nanomaterials with large surface areas and surface-hyperactivity, favorable for drug vectors. In the field of cancer nanomedicine research, biocompatible nanoparticles, such as liposomes and biodegradable polymers, can be loaded with conventional chemotherapeutic drugs, especially those with low solubility, to achieve a controlled drug release profile [16]. Nanovectors facilitate the biodistribution of drugs and improve the bioavailability. The encapsulation efficiency of nanoparticles can be optimized by controllable components and formulation parameters [17,18]. Due to their small sizes, these nanovectors carrying anti-cancer drugs could penetrate into the tissue and passively target tumor tissues by the enhanced permeability and retention (ERP) effect [19]. Recently, great progress has been made in the blood-brain barrier-crossing nanotechnology, and multifunctional nanoplatforms have been constructed to provide highly efficient brain cancer theranostics [20]. Furthermore, nanomaterials have been reported to effectively reverse tumor resistance compared to conventional cytotoxic drugs by stimulating the endocytosis of drug-resistant cells or prohibiting drug efflux with an increase in the drug therapeutic concentration [21]. The constructed nano-drug delivery platforms by mesoporous silica nanoparticles, carbon nanotubes and calcium phosphate nanoparticles, etc. exhibited an enhanced efficiency in delivering the therapeutic agents to desired tumors sites [22]. Large surface areas and surface hyperactivity make nanoparticles apt to surface modifications. By conjugating with the specific tumor markers on the surface of nanovehicles, target delivery of anti-cancer drugs can be realized. A typical example is theranostic nanoparticles which are usually externally-conjugated with antibody and polymers for targeting purposes, and internally-loaded with different bioactive molecules (nucleic acids, imaging contrast agents, etc.) for functional purposes [23,24].

Among these diversified nanomaterials, functionalized fullerenes have attracted substantial attention owing to their various functions in cancer diagnosis and therapy [25]. Their empty interior cavity allows the encapsulation of metal atoms, thus assisting in realizing the electronic basis needed for magnetic resonance imaging (MRI). However, the unique chemical structure renders fullerene to readily generate ROS by light exposure, offering the basis for photodynamic therapy [26,27]. More recently, a novel amine group-modified fullerene derivative (C70-(EDA)8) was reported to have the potential to fight against superbacteria owing to its unique electrostatic and hydrophobic interactions with the membrane of multidrug-resistant superbacteria [28]. The studies by Wang et al. demonstrated that gadofullerenol (C70-OH) nanoparticles could alleviate the acute lung injury by simultaneously scavenging free radicals and modulating pulmonary fibrosis-associated TGFβ signaling [29]. More interestingly, C60-carboxyfullerene has been reported to act as peroxidase mimetics and monitor the glucose level in serum [30]. Therefore, the family of fullerenes possesses diversified medical functions and has shown great promise in biomedical applications.

Gd@C_82_(OH)_22_, a novel metallofullerenol first reported by our group, has been demonstrated to possess an efficient anti-tumor activity with free-carry of other anti-cancer drugs. Further, substantial studies by other scientists also confirmed the anti-tumor potentials of Gd-metallofullerenol [31,32]. More importantly, no obvious toxicity has been observed in animal model experiments, which can be attributed to the fact that Gd@C_82_(OH)_22_ inhibited tumors by imprisoning rather than poisoning the tumor cells. Gd@C_82_(OH)_22_ has shown great potential to resolve the direct cytotoxicity facing traditional chemotherapy. However, tumor encaging by Gd@C_82_(OH)_22_ also suggests its great advantages as a neoadjuvant therapy, which has caused increasing attention in clinical practice. Neoadjuvant therapy, usually as a preoperative strategy, has been widely adopted in the treatment of various cancers [33,34,35]. Especially for certain cancer types with highly-metastatic potentials, the adoption of surgery probably receives unsatisfactory outcomes in clinical settings. Encaging tumor by Gd@C_82_(OH)_22_ treatment in advance of surgery is speculated to greatly increase the operability of surgery and improve prognosis. Herein, Gd@C_82_(OH)_22_ may be an attractive option for neoadjuvant therapy against tumors. In summary, tumor encaging by Gd@C_82_(OH)_22_ is of great clinical significance.

## 2. Gd@C_82_(OH)_22_ as a Novel Anti-Tumor Drug Candidate

### 2.1. Physicochemical Characteristics of Gd@C_82_(OH)_22_

Gd@C_82_(OH)_22_ is a novel endohedral metallofullerenol compound with a unique structure and surface characteristics. Structurally, the Gd atom is encapsulated in a C_82_ carbon cage and the surface of carbon cage is modified with approximately 22 hydroxyl groups [36]. Hydroxyl groups are distributed asymmetrically on the carbon cage [37]. The biased distribution of hydroxyl groups on one side of carbon cage induces more exposure of the hydrophobic carbon cage on the other side. Thus, a single Gd@C_82_(OH)_22_ molecule exhibits amphipathicity with hydrophilic OH groups and hydrophobic cage surface. However, considering that only one fourth of the carbon atom is functionalized with hydroxyl groups, the Gd@C_82_(OH)_22_ molecule is largely hydrophobic. Thus, Gd@C_82_(OH)_22_ molecules tend to aggregate into clusters with the hydration diameter of 70 nm in water or PBS. The formed clusters have been stable, as shown by a single peak size distribution in a physiological medium using dynamic light scattering analysis [38].

Furthermore, the polyhydroxylation of metallofullerenes can direct the electron transfer between the innermost Gd and the outer carbon cage, making the outer carbon cage negatively-charged and the inner Gd positively-charged [37]. Thus, the charge distribution of Gd@C_82_(OH)_22_ may influence its interactions with biomolecules which have differently-charged residues protruding on the surfaces.

### 2.2. Anti-Tumor Activity with High Efficiency and Low Toxicity

Gd@C_82_(OH)_22_ has been intensively investigated ranging from chemical synthesis, property investigation, biological effects and the potential safety concerns [36,38,39]. It has been demonstrated to inhibit tumor growth and metastasis with high efficiency and low toxicity in vitro and in vivo [40,41,42].

In the mice subcutaneously implanted with H22 hepatoma cells, Gd@C_82_(OH)_22_, administrated by intraperitoneal injections, inhibited tumor growth much more efficiently than the conventional first-line chemotherapeutics. Gd@C_82_(OH)_22_ showed a comparable efficiency in inhibiting tumor growth as cyclophosphamide that was used at a 20 times higher dosage [26]. Similarly, in the nude mice bearing breast cancer, the dosage of Gd@C_82_(OH)_22_ used to achieve a comparable tumor inhibition was just one third of the paclitaxel dosage [36]. In addition to tumor growth suppression, Gd@C_82_(OH)_22_ was also found to inhibit tumor metastasis. As shown in a tumor invasion model, a continuous 6-week treatment of Gd@C_82_(OH)_22_ significantly prevented the translocation of breast cancer cells (MDA-MB-231) and the consequent tumor metastasis, whereas the establishment of tumor foci were apparently observed in the saline-treated control mice [43].

Apart from efficacy, the potential toxicity of drug candidates is viewed as another important determinant factor that governs clinical applications. Excitingly, Gd@C_82_(OH)_22_ induced no obvious toxicity, quite distinct from the conventional chemotherapeutic agents. First, Gd@C_82_(OH)_22_-treated mice survived well with no animal deaths occurring, while the paclitaxel- and saline-treated groups had animal deaths. Second, Gd@C_82_(OH)_22_-treated mice had undetectable weight alterations compared with the obvious weight loss in paclitaxel-treated mice. Third, paclitaxel treatment worsened tumor-induced nephrotoxicity marked by irreversible glomerulitis and cell necrosis. Instead, Gd@C_82_(OH)_22_ induced no toxicity in important organs and even alleviated tumor-induced damage in kidney functions [27,42]. Last, no cytotoxicity was induced during testing by Gd@C_82_(OH)_22_ in a variety of cell lines, including normal cells and tumor cells.

Therefore, Gd@C_82_(OH)_22_, with a much lower dosage, exhibited a comparable tumor inhibition to the conventional chemotherapeutic drugs with marginal side effects, indicating the overwhelming advantages of Gd@C_82_(OH)_22_ as a promising anti-tumor drug candidate.

### 2.3. A Novel Anti-Tumor Mechanism by Gd@C_82_(OH)_22_: Encaging Tumors

**Figure 1:**
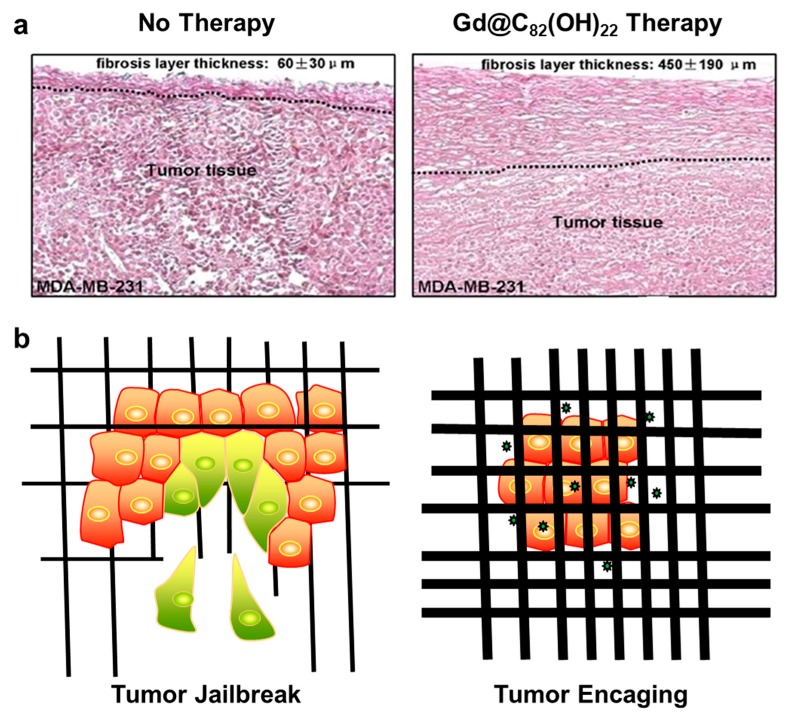
Tumor encaging by Gd@C_82_(OH)_22_ therapy. (**a**) Histological images of tumor tissues excised from Gd@C_82_(OH)_22_-treated mice and the saline control mice. The tumor tissues from control (**Left**) and Gd@C_82_(OH)_22_-treated (**Right**) MDA-MB-231 xenograft mice; A thicken fibrosis layer surrounding tumor tissues was formed in Gd@C_82_(OH)_22_-treated animals. Reproduced with permissions from Meng, H, Nanomedicine; published by Elsevier Inc. 2012. (**b**) A sketch for tumor encaging by Gd@C_82_(OH)_22_. Tumor jailbreak (**Left**). The tumor cells secrete a large amount of matrix metalloproteinases (MMPs) to degrade the extracellular matrix and realize self-escape. Gd@C_82_(OH)_22_ therapy inhibits tumor invasiveness and metastasis by tumor encaging (**Right**) (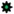 represents Gd@C_82_(OH)_22_ molecule).

To date, anti-tumor multi-mechanisms have been identified for Gd@C_82_(OH)_22_ including immune modulation [44,45], ROS scavenging [46], angiogenesis inhibition [42], fibroblast deactivation [47], iron uptake inhibition [48], the suppression of cancer stem cells [49] and tumor imprisonment or encaging [43]. Among these mechanisms, tumor encaging is a novel mechanism first reported by our group. In the mice implanted with human breast cancer cells, a thick fibrous layer on tumor surface was formed in Gd@C_82_(OH)_22_-treated mice. The thickness of the dense connective tissue surrounding the tumor was determined to be 450 μm. Conversely, a natural tumor extracellular matrix with an average thickness of 60 μm was formed at the tumor boundary in saline-treated control mice (Figure 1a). Moreover, the fibrous layer was undetectable in other organs, suggesting that the formation of fibrous layer induced by Gd@C_82_(OH)_22_ treatment was limited to tumor tissue with the absence of the general fibrosis [42]. The authors postulated that the formed thick fibrous cage may encapsulate cancer tissue, and the communications between the cancer and surrounding tissues was cut so that the tumor was imprisoned. A sketch of tumor encaging by Gd@C_82_(OH)_22_ is depicted in Figure 1b. Imprisoning rather than directly poisoning tumor cells accounted for the low toxicity of Gd@C_82_(OH)_22_ in anti-tumor effects, at least partially.

### 2.4. Molecular Mechanism of Tumor Encaging: MMP Inactivation and Collagen Stabilization

**Figure 2:**
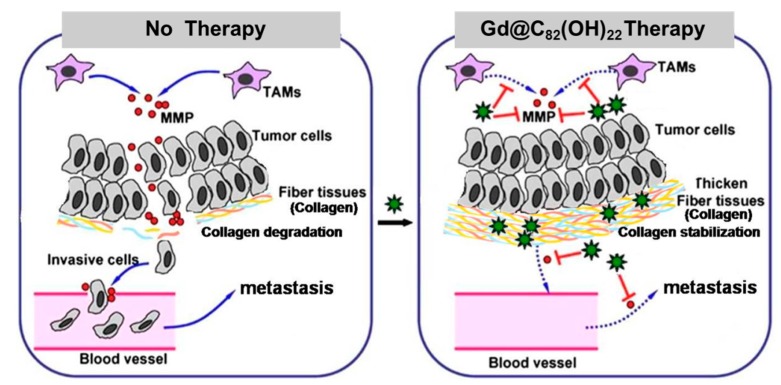
Schematic presentation of tumor encaging by Gd@C_82_(OH)_22_. Instead of directly poisoning tumor cells, Gd@C_82_(OH)_22_ encaged tumor via MMP inhibition and collagen stabilization. In the control mice (**left** panel), MMP secreted by tumor-associated macrophages (TAMs) efficiently degraded the collagen-consisting fibrous matrices. Consequently, tumor cells escaped and tumor metastasis occurred. In Gd@C_82_(OH)_22_-treated mice (**right** panel), Gd@C_82_(OH)_22_ decreased MMP production and activity and furthermore stabilized collagen by facilitating the formation of collagen microfibrils. The formed thick fibrous layers imprisoned tumor cells and the tumor failed to metastasize. (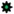 represents Gd@C_82_(OH)_22_ molecule). Adapted with permissions from Meng, H, Nanomedicine; published by Elsevier Inc. 2012.

As described in Figure 2, MMP deactivation and collagen stabilization contributed to tumor encaging by Gd@C_82_(OH)_22_. On the one hand, Gd@C_82_(OH)_22_ inhibited the matrix-degrading function of MMPs and thus prevented the proteolytic degradation of extracellular matrix in tumor progression [42]. On the other hand, Gd@C_82_(OH)_22_ acted as a bridge to facilitate the formation of collagen oligomers or microfibrils, inducing the enhanced stability and rigidity of collagen layers.

By molecular-dynamics simulations, the interactions of Gd@C_82_(OH)_22_ with MMPs and collagen were investigated. Gd@C_82_(OH)_22_ was found to inhibit MMP-9 functions in an exosite way by allosteric regulation rather than targeting the zinc-binding active sites. Before approaching MMP-9, Gd@C_82_(OH)_22_ clustered and formed a trimer step by step relying on molecular interactions. Owing to the negative electric field that formed around the zinc-binding sites, the negatively-charged carbon cages were repelled, thus impeding the bindings to zinc-binding active sites. The negatively-charged carbon cages were guided to approach MMP-9 by two positively-charged residues (Lys 433 and Arg 440) via long-range electrostatic interactions, allowing the initial contact of Gd@C_82_(OH)_22_ with MMP-9. Afterwards, this interaction between Gd@C_82_(OH)_22_ clusters and these two residues were facilitated by the formation of hydrogen bonds between the hydroxyl groups on carbon cages and the basic groups of residues. Following this, the hydrophobic interactions occurred between the largely hydrophobic surface of Gd@C_82_(OH)_22_ and the hydrophobic residues exposed on the surface of the MMP-9 molecule. Finally, Gd@C_82_(OH)_22_ stably bound the sites near the S1 ligand specificity loop and SC loop by a combination of both specific electrostatic and hydrophobic interactions, probably prohibiting the substrate entry into the S1 ligand specificity loop [50]. Therefore, it seemed that the inhibition mode of MMP-9 by Gd@C_82_(OH)_22_ was quite distinctive, depending on the unique physicochemical characteristics of Gd@C_82_(OH)_22_.

As known, collagens, as one of major ECM components, are also tightly related with tumor progression [51]. Their expression, proteolysis and assembly all influence cellular functions to elicit multiple effects on tumors [52]. Gd@C_82_(OH)_22_ has been reported to adhere to and interact with collagen molecules principally depending on the interaction of hydrogen bonds. The negatively-charged carbon cage was more preferentially bound to the positively-charged N-terminus of collagen peptide chains where the strong electrostatic interactions happened with the largest number of the adhering Gd@C_82_(OH)_22_ molecules. After adhering on the surface of collagen peptide chains, the hydrogen bonds formed between Gd@C_82_(OH)_22_ and collagen peptide chains, thus facilitating the stability of the collagen triplex structure. Furthermore, Gd@C_82_(OH)_22_ promoted the formations of collagen oligmers and microfibrils by hydrogen bonds’ interaction with multiple polypeptide chains of collagen triplex, which the authors called fullerenol-mediated bridge [53]. Therefore, by electrostatic or hydrogen bonds interaction with collagen molecules, Gd@C_82_(OH)_22_ stuck and stabilized the collagen molecules, rendering collagen less susceptible to degradation by MMPs. Gd@C_82_(OH)_22_-induced collagen crosslinking provided another basis for the thicken fibrous layers formed surrounding the tumor tissues.

## 3. An Inspiration for Novel Drug Design: The Advantages of Nanomedicine

### 3.1. Exploitation of Tumor Imprisonment: The Development of MMP Inhibitors

As known, both tumor growth and metastasis are largely dependent on the remodeling of the extracellular matrix which serves as physical supports to confine tumor cells [54,55]. To perform a jailbreak, tumor cells produce and secrete a large amount of matrix-cleaving enzymes directly. More often, stromal cells, such as tumor-associated fibroblasts, are recruited by the tumor and assist tumor cells to escape by secreting MMPs [56]. MMPs assist the tumor to cleave the surrounding extracellular matrix. Thus tumor cells escape from ECM imprisonment and finally invade other tissues more malignantly. Accumulating evidence has highlighted the tight associations between high-level MMPs and tumor malignancy [57,58]. In tumor tissues, MMPs are usually up-regulated, advantageous to tumor growth and metastasis [59]. Hence, MMPs are believed to hold the promise as molecular target of cancer therapy.

### 3.2. Failure of Broad-Spectrum MMP Inhibitors: The Dilemma of Classical Drug Design

Preventing ECM degradation by MMPs is considered as a new route to fight cancer. In the past two decades, many pharmaceutical companies have made great efforts to develop many MMP inhibitors [60]. Unexpectedly, these MMP inhibitors were disappointing in clinical trials, either with no efficacy or with severe adverse reactions [61]. The key reason for the failure lies in the lack of selectivity towards the specific MMPs by the designed MMP inhibitors [62]. In fact, the substrates of these MMPs are much more than extracellular matrix and different MMP isoforms play diversified roles in different stages of the tumor [63]. By proteolysis, some of the MMP isoforms can activate growth factors, for instance, angiostatin that inhibits tumor angiogenesis and metastasis [64]. The broad-spectrum MMP inhibitors target all MMPs, even those tumor-suppressing MMP members. This tribulation calls for a new generation of MMP inhibitors with enhanced selectivity and specificity. However, chemically, it is challenging to design the highly specific MMP inhibitors due to high similarities in MMPs’ active sites. In fact, low selectivity is very common to small-molecular drugs which are traditionally designed according to the key and lock principle [65]. The key and lock principle, firstly put forward based on the model for enzyme-substrate interaction, mainly focuses on the active sites of enzymes. The specific complementary geometric shapes are suggested to be possessed by enzyme and substrate. Like a key into a lock, only the correct size and shape of the substrate (the key) could fit into the active site (the keyhole) of the enzyme (the lock). Following this, the key and lock principle extends its application to drug design. The designed small molecule drugs, with the correct geometric shapes and chemical properties, occupy the active sites and impact the function of biomolecules and ultimately disturb the associated biological process [66]. Despite the success in conducting the development of some molecule-targeted drugs, this canonical principle also encounters the dilemma where off-target occurred or low selectivity is very common. By strong chemical bonds, the interaction between small-molecular drugs and the active sites, in most instances, is compounded by the presence of high similarities in active sites possessed by a family of proteins and generated indiscriminate inhibitions. Therefore, a new strategy of drug design is urgently expected.

### 3.3. Action Mode of Gd@C_82_(OH)_22_ Towards Key Biomolecules: The Advantages of Nanomedicine

The structure-activity relationship where the structures of drug molecules affect their interactions with biomolecules and the sequent biological functions is widely recognized as the basis of drug design [67]. The structure-activity relationship should be elaborated when it comes to discussions on the advantages of nanomedicine:

First, the chemical structure impacts the stability of nanomedicine and the physiochemical properties. The authors previously discussed the impacts of surface-chemical design on physiochemical properties and biological effects of various carbon nanomaterials including carbon nanotubes, graphenes, fullerenes, metallofullerenes and their derivatives in detail. The reader may refer to Ref [37]. Our present discussion will be focused on metallofullerenols. The chemical modification on metallofullerene surface is highly related with the opening of unsaturated double bonds on the carbon cage and the addition of chemical groups onto the surface. Thus, to maintain the stability and integrity of carbon cage, the number of added chemical groups has to be controlled. For multihydroxylated fullerene, the total number of modified groups on the cage surface should be controlled in order not to destroy the carbon cage arising from the strong interaction between the neighboring hydroxyls. The modification of 22 hydroxyl group with −OH number varying in ±2, was verified to have a favorable chemical stability.

Apart from the impacts on the structural stability, the surface chemistry of fullerene cage can also modulate the electronic configuration of the encaged metallic atoms inside a nanospace and the electron donation directions, thus influencing the electronic and magnetic properties of metallofullerene. Using synchrotron X-ray photoelectron spectroscopy, the authors previously investigated the modulations of electronic configurations of the innermost Gd atoms inside different nanospaces provided by Gd@C_82_, Gd@C_82_(OH)_12_, Gd@C_82_(OH)_22_. A sandwich-type electronic interaction occurred in a synergistic manner along the pathways. The cage modification, group-cage, surfaced the innermost metallic atom [68]. The hydroxyl number also affected the electronic properties and electron-transfer direction [37,69]. The electron emission showed a periodical variation depending on the number of added hydroxyl groups on the outer cage surface. It provides a possibility to control electronic donation direction by surface chemical modifications [68].

As previously discussed, both the number of modified groups and their distribution patterns on the outer carbon surface are the key factors determining the modulation of nanomaterial structures and the corresponding properties of fullerene derivatives. The multihydroxylation increased the solubility of Gd@C_82_(OH)_22_ in water. The nanosize and the more hydrophobicity rendered Gd@C_82_(OH)_22_ readily to aggregate with the size of 22 nm. The structure of Gd@C_82_(OH)_22_, as stated above, allows the formation of hydrogen bonds and hydrophobic interaction, respectively. The negatively-charged carbon cage tends to cause electronic interaction with the positively-charged molecules.

**Figure 3:**
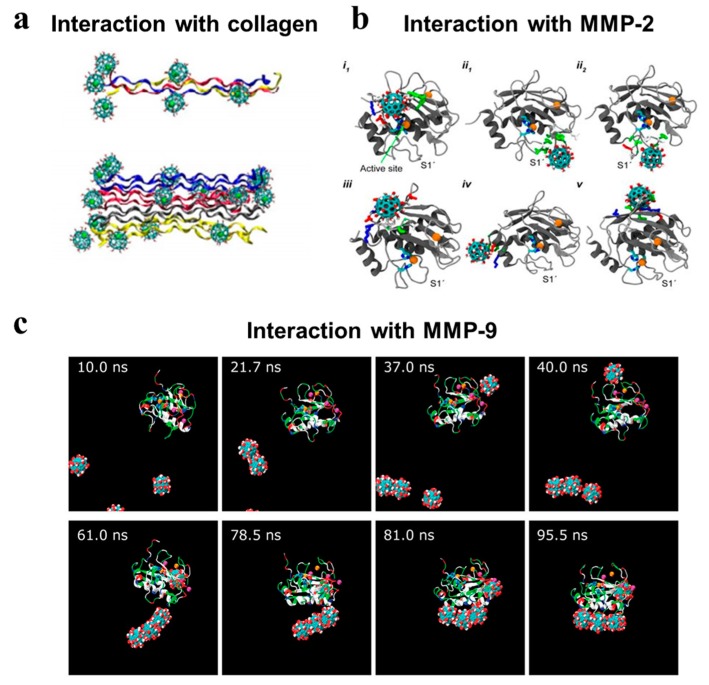
Molecular dynamics of Gd@C_82_(OH)_22_ interacting with key ECM-associated biomolecules. Gd@C_82_(OH)_22_ could bind to collagen and MMPs step by step but differ in the binding modes. (**a**) Gd@C_82_(OH)_22_ interacting with collagen molecules. Gd@C_82_(OH)_22_-mediated bridge among collagen molecules greatly restricted their relative rotation and crosslinked collagen molecules [53]. (**b**) Gd@C_82_(OH)_22_ interacting with MMP-2. Gd@C_82_(OH)_22_ could block Zn^2+^-catalytic site directly or exocitely bind at the ligand specificity S1’ loop [75]. (**c**) Gd@C_82_(OH)_22_ interacting with MMP-9. Gd@C_82_(OH)_22_ induced a specific binding with MMP-9 near the ligand-specificity S1’ loop [50]. Reproduced with permissions from Yin. X.H, Nanoscale; published by The Royal Society of Chemistry, 2013. Kang S.G, Scientific reports; published by Nature Publishing Group, 2014. Kang S.G, Proceedings of the national academy of sciences; published by Natl Acad Sciences, 2012.

Second, the structure of nanomedicine affects its interactions with biomolecules. The interaction modes of nanomedicine with biomolecules can be manipulated by nano-scaled size and the controllable chemical modifications, which probably endow drug designs with high feasibility and flexibility. While different from the small-molecular drugs interacting with target molecules via strong chemical bonds, nanomedicine interacts with different biomolecules in a much milder and weaker manner, mainly via electrostatic, hydrophobic and specific hydrogen-bonding interactions. These interactions may direct nanoparticles to cluster into different shapes and guide the binding of nanoparticles to the specific surface region of biomolecules, making nanoparticles target-tailoring. As active sites are often conservative in a biomolecule family, regulatory domains usually bearing a much lower similarity, tend to be a more promising alternative for the drug design. Gd@C_82_(OH)_22_ inhibited MMP-9 mainly via allosteric regulation of an exocite interaction with little involvement of the well-known zinc catalytic site [50]. Gd@C_82_(OH)_22_ interacted with the approaching biomolecules MMP-9 or collagen depending on different cage positions to contact with certain biomolecules. The interactions of Gd@C_82_(OH)_22_ with other biomolecules also provide support for this specific action mode of Gd@C_82_(OH)_22_ based on its unique structural characteristics [70,71,72]. Figure 3 demonstrates the results on molecular dynamics of Gd@C_82_(OH)_22_ interacting with key ECM-associated biomolecules including collagen, MMP-9 and MMP-2, indicating the selective and specific binding mode of Gd@C_82_(OH)_22_ with biomolecules. Recently, Zhou et al. substituted a new functional group, PO_4_^2-,^ for a hydroxyl group on the fullerenol surface of Gd@C_82_(OH)_22_ and then found that Gd@C_82_(OH)_21_(PO_4_)^2-^ bound more strongly to MMP-9 than Gd@C_82_(OH)_22_ [73]. More importantly, it is worth emphasizing that the favorable weak interactions may still maintain the native fold of target proteins, which accounts for the slight toxicity induced by MMP inhibition after Gd@C_82_(OH)_22_ treatment. Contrary, the strong hydrophobic and aromatic stacking interactions of the single-wall carbon nanotubes (SWCNTs) was reported to destroy the protein hydrophobic core and tertiary structure, which led to obvious nanotoxicity [74]. Therefore, it can be tentatively inferred that the formed shape of nanoclusters and the contact parameters with biomolecules including the size of contact areas, the hydrophobic or hydrophilic contact sites and the intensity of interactions, can be manipulated by controlled particle sizes and surface modifications, which influences the binding specificity and selectivity toward certain target molecules.

Third, the structure of nanomaterials is a key determinant factor in the biological effects of nanomaterials. Previously, the authors compared the toxicity of Gd@C_82_(OH)_22_ and other carbon nanomaterials to illustrate the role of the structure in determining biological effects. For example, after respiratory exposure to the single-wall carbon nanotubes (SWCNTs) for 72 h, the spontaneously hypertensive rats developed pulmonary inflammation and peripheral vascular thrombosis [76]. Carbon nanotubes were observed to destroy the membranes of human gut bacteria [77]. Moreover, the cytotoxicity of carbon nanomaterials with different structures was compared, including single-wall nanotubes (SWNTs), multi-wall nanotubes (MWNTs) and fullerene (C_60_) on alveolar macrophages, and C_60_ was found to induce the smallest cytotoxicity [78]. Later, the biological effects of fullerene derivatives Gd@C_82_(OH)_22_ and C_60_(OH)_20_ were compared, especially the anti-tumor activity. Resultantly, C_60_(OH)_20_ was found to possess an attenuated anti-tumor activity compared with Gd@C_82_(OH)_22_, which was finally tracked to the differences in the number and distribution pattern of hydroxyl groups on the carbon cage of these two different fullerene derivatives [79]. A smaller number of hydrogen bonds formation happened when C_60_(OH)_22_ interacted with collagen peptide triplex. Compared with C_60_(OH)_22_, Gd@C_82_(OH)_22_ had a smaller percentage of carbon atoms functionalized by hydroxyl groups and exposed more of the carbon cage surface, being more hydrophobic. The negatively-charged fullerenol cage facilitated the electrostatic interaction with collagen. The stronger hydrophobic and electrostatic interaction facilitated the absorption of Gd@C_82_(OH)_22_ on collagen molecules, allowing a more favorable formation of hydrogen bonds with collagen molecules.

**Figure 4:**
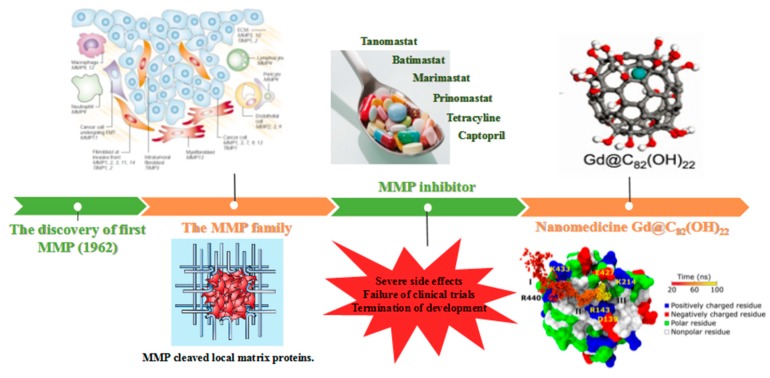
A brief history of MMP-based discovery: From MMP inhibitors to Gd@C_82_(OH)_22_. After the discovery of the first MMP in 1962 [80], the intercellular regulation of MMP family was also explored, particularly in tumor cells [56,81]. Subsequently, the development of MMP inhibitors was initiated to achieve tumor confinement. However, due to their low selectivity and severe side effects, these MMP inhibitors failed in clinical trials and drug development was abandoned. The emergence of Gd@C_82_(OH)_22_ may bring the light for the development of selective MMP inhibitors owing to the unique physicochemical characteristics of nanomaterials [42,50]. The figure is constructed based on the above references. Adapted with permissions from Egeblad. M, Nature reviews cancer; published by Nature Publishing Group, 2002, Kang S.G, Proceedings of the national academy of sciences; published by Natl Acad Sciences, 2012. Yamada K.M, Nature; published by Nature Publishing Group, 2003. Meng H, ACS Nano; published by American Chemical Society, 2010.

Due to the flexibility and diversity of interacting with certain molecules, nanomedicine, such as Gd@C_82_(OH)_22,_ seems to be a transformable and dynamic key that could match several locks depending on the surface characteristics of the target biomolecules. In detail, the canonical key and lock principle requires the existence of the complementary geometric shapes between the molecular drug (the key) and the target biomolecules (the lock) to fit into the active site (the keyhole). If the molecular drug cannot match the active site of the target biomolecules, off-target happens and no drug efficacy generates. In contrast, designed nanomedicine could intelligently detect the surface characteristics and selectively interact with the unique surface area of target biomolecules, thus enhancing the target specificity. Depending on the surface characteristics of the target biomolecules, nanomedicine, just like a transformable and dynamic key, can self-assemble into suitable shapes to match several locks for thermodynamic stability, suggesting the target-tailoring ability of nanomedicine. Figure 4 demonstrates a brief history of MMP-based research from the discovery of the first MMP in 1962 [80], the consecutive identifications of MMP family members and their biological functions [81], the development of MMP inhibitors to novel MMP-inhibiting nanomedicine Gd@C_82_(OH)_22._

## 4. Conclusions and Perspectives

Despite being in its infancy, nanomedicine, due to its unique physical-chemical properties, holds great promise in the near future. As Gd@C_82_(OH)_22_ starts the journey to clinical application, a new antineoplastic mechanism of imprisoning rather than poisoning the tumor has been uncovered, which may supply solutions for alleviating or conquering the toxicity faced by traditional chemotherapy. Furthermore, the action mode of nanoparticles with biomolecules has been revealed, implicating the de novo design of drugs and the potential of nanotechnology in revolutionizing the current strategy of drug design.

However, challenges also exist for Gd@C_82_(OH)_22_ to step forward for clinical applications. The definite distribution analysis of hydroxyl groups on the carbon cage have to be identified. Moreover, the identification of metabolites after Gd@C_82_(OH)_22_ entering the body is another important issue to be addressed, which reflects the behaviors of Gd@C_82_(OH)_22_ in the body and influences its toxicological profile. In spite of the clinical challenges facing Gd@C_82_(OH)_22_, Gd@C_82_(OH)_22_, as a promising novel anti-tumor nanomaterial, exhibited a highly-efficient antineoplastic activity with low toxicity based on numerous solid preclinical data. Enormous effort has to be made to translate Gd@C_82_(OH)_22_ from a laboratory innovation to the clinic. Due to the advantages of nanotechnology, society will benefit from nanomedicine in the near future.
